# Centralized Multi-Sensor Square Root Cubature Joint Probabilistic Data Association

**DOI:** 10.3390/s17112546

**Published:** 2017-11-05

**Authors:** Yu Liu, Jun Liu, Gang Li, Lin Qi, Yaowen Li, You He

**Affiliations:** 1 School of Electronic and Information Engineering, Beihang University, Beijing 100191, China; 2 Research Institute of Information Fusion, Naval Aeronautical and Astronautical University, Yantai 264001, China; 3278pirate@163.com (L.Q.); heyou_f@126.com (Y.H.); 3 Department of Electronic Engineering, Tsinghua University, Beijing 100084, China; gangli@mail.tsinghua.edu.cn (G.L.); lyw17@mails.tsinghua.edu.cn (Y.L.)

**Keywords:** multi-sensor tracking, data association, cubature Kalman filter, state estimation, centralized filtering

## Abstract

This paper focuses on the tracking problem of multiple targets with multiple sensors in a nonlinear cluttered environment. To avoid Jacobian matrix computation and scaling parameter adjustment, improve numerical stability, and acquire more accurate estimated results for centralized nonlinear tracking, a novel centralized multi-sensor square root cubature joint probabilistic data association algorithm (CMSCJPDA) is proposed. Firstly, the multi-sensor tracking problem is decomposed into several single-sensor multi-target tracking problems, which are sequentially processed during the estimation. Then, in each sensor, the assignment of its measurements to target tracks is accomplished on the basis of joint probabilistic data association (JPDA), and a weighted probability fusion method with square root version of a cubature Kalman filter (SRCKF) is utilized to estimate the targets’ state. With the measurements in all sensors processed CMSCJPDA is derived and the global estimated state is achieved. Experimental results show that CMSCJPDA is superior to the state-of-the-art algorithms in the aspects of tracking accuracy, numerical stability, and computational cost, which provides a new idea to solve multi-sensor tracking problems.

## 1. Introduction

The centralized state estimation method for multiple-target tracking with multiple sensors can integrate detecting information from different sensors according to certain rules, which will make the tracking results more accurate than that of a single sensor [1,2,3]. Multi-sensor fusion is an important data processing method in the field of target tracking, especially under nonlinear conditions, and the fused result with multiple sensors is often better for multiple-target tracking, which has been widely concerned by scholars both at home and abroad [3,4,5,6,7,8,9,10,11,12].

The state-of-the-art techniques for multiple-target tracking mainly contains two categories. The first category is the tracking method based on a random finite set; the other is the method based on measurement to track association. The former method can directly track multiple targets without associating the detected measurements with the tracks in the surveillance area. However, this kind of method faces complex integral operations, which are often difficult to find an exact solution. Although approximation methods have been a good choice for this problem, the computational cost is too great, and improper approximation will lead to degradation of the tracking accuracy, thus making it a long way from being extensively utilized in practical applications [4]. The latter method has been widely applied in various tracking systems, and is still the most common method for centralized tracking [5,6,7,8,9,10,11,12,13,14].

In the second category, the sequential multi-sensor joint probabilistic data association algorithm (MSJPDA) is a classical and effective method for multi-sensor tracking, which achieves better tracking performance compared with its parallel counterparts [10,11]. Unfortunately, current MSJPDA algorithms are often proposed to solve association problems under linear circumstances, and the nonlinear environment is seldom involved in the literature. As is known, the nonlinear equation can be linearized according to Taylor expansion, then a MSJPDA algorithm based on the extended Kalman filter (EKF), the so called MSJPDA-EKF is derived [12,13,14]. However, it is essential for MSJPDA-EKF to calculate the Jacobian matrix in the linearization step in each iteration, and the ignorance of higher orders may introduce large linearization errors, which may result in degraded performance, and even divergence [1,2,14]. In addition, the estimated state will affect the calculation of association probability in the next iteration, thus causing changes in the computation of weights of joint events, which may eventually makes false correlations, and even lead to divergence [15]. To conquer the shortcomings of MSJPDA-EKF, the MSJPDA algorithm based on the unscented Kalman filter (MSJPDA-UKF) is proposed. MSJPDA-UKF can acquire higher estimation accuracy than MSJPDA-EKF, and is relatively more stable [16]. However, reasonable adjustments are needed in MSJPDA-UKF to achieve the ideal performance, and a numerical instability phenomenon is easy to occur in the situation of high dimensional estimation [16,17,18].

To address the high dimensional nonlinear estimation problem, the cubature Kalman filter (CKF) based on the third-degree spherical-radial cubature rule has been recently proposed [19,20,21,22,23,24,25]. In the procedure of CKF, a certain number of typical cubature points are selected to approximate the posterior probability, then the mean and covariance are captured through a nonlinear transfer function to achieve accurate estimation of the target state [19]. CKF and UKF are both moment-matching filters which deterministically select a set of weighted sample points to approximate the posterior probability density. Compared with EKF, CKF and UKF do not have to calculate the Jacobian matrix in each iteration, thereby reducing the computational cost. At the same time, they both avoid the negative influence of the truncation error in the process of nonlinear state estimation, which is particularly effective in the case of state estimation with strong nonlinearity [19,21,22,23]. In UKF, to achieve a better estimation performance, the adjustment of the scaling parameter is an essential step, as different scaling parameters may yield completely distinct estimation results. The selection of the scaling parameter is of great significance to the performance of UKF in some sense [16,19,21,25]. However, there is no parameter to be adjusted for CKF, and the selected sampling points and weights are only related to the dimension of the target state, which can be calculated in advance to reduce the computational complexity. Furthermore, CKF is more accurate with respect to high dimensional estimation, and easy for design and implementation [19,21,22,23,24]. Especially in the case of high dimension, EKF and UKF suffer from the curse of dimensionality or divergence, or both, but CKF still performs well [19,21,25]. Unfortunately, in practice, arithmetic operations performed on finite word-length digital computers may introduce large errors; then the symmetry and positive definiteness of the covariance are often not satisfied. Moreover, perhaps the loss of positive definiteness is more perilous as it terminates the running of the CKF. Then, the square root version of CKF, the so called SRCKF is proposed to address this problem [19,21,23,24]. In SRCKF, only the square root factors are propagated during the iterations. Therefore, the square rooting operations of the matrix are avoided, thus reducing the computation cost. The symmetry and positive definiteness of the covariance are preserved, and the numerical accuracy is also improved [19,21,25].

In this paper, to deal with the target tracking problem in a nonlinear system under a cluttered environment, a novel centralized multi-sensor square root cubature joint probabilistic data association algorithm (CMSCJPDA) is proposed. In CMSCJPDA, measurements of each single sensor are sequentially processed to compute the association probabilities based on the similar rules in JPDA, then SRCKF and the validated measurements are chosen to estimate the state of targets according to weighed state fusion. Eventually, the accurate estimation of multiple targets’ states with multiple sensors is accomplished.

The rest of this paper is organized as follows: Section 2 describes the problem of tracking multiple targets with multiple sensors. Then, in Section 3, a simple overview of the numerical integral approximation method based on spherical-radial principle is introduced. The main idea of CMSCJPDA is detailed in Section 4, and an algorithm flow is given. The numerical experiments are designed in Section 5, and the comparison and analysis of CMSCJPDA against several existing methods is also presented in this section. Concluding remarks of this paper and future work are given in Section 6.

## 2. Problem Formulation

Consider sensors are applied to track $N_{t}$ targets in a cluttered environment. For arbitrary target *t* $\left(1\leq t\leq N_{t}\right)$, $X^{t}\left(k\right)$ denotes the state of target *t* at discrete time instant *k*. For brevity, without the control input term, the discrete-time state equation of a nonlinear system is:

(1) $$X^{t}\left(k+1\right)=f^{t}\left[k,X^{t}\left(k\right)\right]+V^{t}\left(k\right)$$

where *k* is the discrete time instant, $f^{t}\left(\cdot \right)$ is a known nonlinear function, and $V^{t}\left(k\right)$ is independent zero-mean Gaussian process noise with covariance:

(2) $$E\left\{V^{t}\left(k\right){\left[V^{t}\left(l\right)\right]}^{T}\right\}=Q^{t}\left(k\right)\delta \left(k,l\right)$$

where δ(k,j) is the Kronecker delta function.

The measurement equation of sensor *i* for target *t* is:

(3) $$Z^{i,t}\left(k\right)=h^{i}\left[k,X^{t}\left(k\right)\right]+W^{i}\left(k\right)$$

where $i=1,2,\cdots ,N_{s}$ represents the label of sensors. $Z^{i,t}\left(k\right)$ is the measurement vector of sensor *i* for target *t* at time instant *k*. $h^{i}\left(\cdot \right)$ represents a known nonlinear function. $W^{i}\left(k\right)$ is the zero-mean, independent of process noise in Equation (3) and independent from sensor to sensor, with covariance:

(4) $$E\left\{W^{i}\left(k\right){\left[W^{i}\left(l\right)\right]}^{T}\right\}=R^{i}\left(k\right)\delta \left(k,l\right)$$

## 3. Numerical Approximation of the Multi-Dimensional Weighted Integral

### 3.1. Third-Degree Spherical-Radial Rule

Before introducing the CMSCJPDA method, a brief description of the numerical integral approximation based on spherical-radial principle is given. Consider the following multi-dimensional Gaussian weighted integral:

(5) $$I\left(f\right)=\int_{R^{n}}f\left(x\right)\exp\left(-x^{T}x\right)dx$$

where f(⋅) is an arbitrary function, $R^{n}$ denotes the integral region. As is known, the key to addressing the problem of nonlinear filtering based on Bayesian theory is to compute the first-order and second-order moments. In other words, the core of the Bayesian filter is how to compute Gaussian weighted integral which is of the form *nonlinear function* × *Gaussian density* that is illustrated in Equation (5).

Let x=ry, where $y^{T}y=1$, So that $x^{T}x=r^{2}$ for r∈[0, ∞). Then the integral Equation (5) can be rewritten as:

(6) $$I\left(f\right)=\int_{0}^{\infty }\int_{U_{n}}f\left(ry\right)r^{n-1}\exp\left(-r^{2}\right)d\sigma \left(y\right)dr$$

where $U_{n}=\left\{y\in R^{n}|y^{T}y=1\right\}$ is the surface of the sphere and σ(⋅) is the spherical surface measure or the area element on $U_{n}$. Therefore, we can split Equation (6) into two integrals:

(7) $$I\left(f\right)=\int_{0}^{\infty }S\left(r\right)r^{n-1}\exp\left(-r^{2}\right)dr$$

(8) $$S\left(r\right)=\int_{U_{n}}f\left(ry\right)d\sigma \left(y\right)$$

where I(f) is the radial weighted integral that is shown in Equation (7), S(r) is the spherical integral with the unit weighting function ω(y)=1 that is shown in Equation (8).

Then the above integrals described in Equations (7) and (8) can be solved according to the third-degree spherical cubature rule and radical rule, respectively:

(9) $$\int_{U_{n}}f\left(y\right)d\sigma \left(y\right)\approx \omega \sum_{i=1}^{2n}f{\left[u\right]}_{i}$$

(10) $$\int_{a}^{b}f\left(x\right)\omega \left(x\right)dx\approx \sum_{i=1}^{m}\omega_{i}f\left(x_{i}\right)$$

where ${\left[u\right]}_{i}$ represents the *i*-th element of generator u, and u is a generator if $u=\left(u_{1},u_{2},\ldots ,u_{r},0,\ldots 0\right)\in {\mathbb{R}}^{n}$, where $u_{i}>u_{i+1}>0,\;i=1,2,\ldots ,\left(r-1\right)$. For simplicity, the (n−r) zero coordinates are ignored and notation $\left[u_{1},u_{2},\ldots ,u_{r}\right]$ is used to represent a complete fully-symmetric set of points that can be obtained by permutating and changing the sign of generator u in all possible ways. For example, $\left[1\right]\in {\mathbb{R}}^{2}$ denotes the following set of points:

(11) $$\left[1\right]=\left\{\left(\begin{array}{c} 1\\ 0 \end{array}\right),\;\left(\begin{array}{c} 0\\ 1 \end{array}\right),\;\left(\begin{array}{c} -1\\ 0 \end{array}\right),\;\left(\begin{array}{c} 0\\ -1 \end{array}\right)\right\}$$

where ω(x) is a known weighting function which is non-negative on the interval [a, b].

As is shown in Equation (9), the spherical cubature rule indicates that the spherical integral can be approximated by a weighted sum of function values at the sample points, which are located at the intersection of the unit sphere and its axes. The radical rule described in Equation (10) indicates that an *m*-point Gaussian quadrature is equal to the sum of (2*m* − 1) polynomials [21].

Especially, a standard Gaussian weighted integral can be computed based on third-degree spherical-radial rule as follows [25]:

(12) $$I_{N}\left(f\right)=\int_{R^{n}}f\left(x\right)N\left(x\;;\;0,I\right)dx\approx \sum_{i=1}^{2n_{x}}\omega_{i}f\left(\xi_{i}\right)$$

where:

(13) $$\xi_{i}=\sqrt{\frac{2n_{x}}{2}}{\left[1\right]}_{i},\;\omega_{i}=\frac{1}{2n_{x}},\;i=1,2,\cdots ,2n_{x}$$

where $\xi_{i}$ is the cubature point, $\omega_{i}$ is the corresponding weight. ${\left[1\right]}_{i}$ denotes the *i*-th row or column of the generator [1].

Actually, the weighting function term ω(⋅) of the integrand is often not subject to the standard Gaussian distribution. In other words, the Gaussian weighted integral in nonlinear filtering cannot be solved directly by Equation (12). Fortunately, the following equation makes it possible to work out the nonstandard Gaussian weighted integrals which occur in nonlinear condition:

(14) $$\int_{R^{n}}f\left(x\right)N\left(x;\;\mu ,\;\Sigma \right)dx=\int_{R^{n}}f\left(\sqrt{\Sigma }x+\mu \right)N\left(x;\;0,\;I\right)dx$$

Through Equation (14), which is proved in Appendix A, the nonstandard Gaussian weighted integrals can be transformed into the standard ones. Then, the approximation of posterior mean and error covariance can be addressed by Equation (12). Thus, an iteration of the time and the measurement updates in the Bayesian filter is completed.

### 3.2. Accuracy Analysis

Assume an *n*-dimensional vector $x∼N\left(\bar{x},\;P\right)$, where $\bar{x}$ is the mean of x, and P is the corresponding covariance. We extend the nonlinear function f(x) at $\bar{x}$ based on Taylor extension:

(15) $$f\left(x\right)=f\left(\bar{x}\right)+D_{\Delta x}f+\frac{D_{\Delta x}^{2}f}{2!}+\frac{D_{\Delta x}^{3}f}{3!}+\frac{D_{\Delta x}^{4}f}{4!}+\cdots$$

where $\Delta x=x-\bar{x}$, $D_{\Delta x}f={\left[\Delta x\right]}^{T}{\nabla f\left(x\right)|}_{x=\bar{x}}$, ∇f(x) is the partial derivative of f(x).

(1) We use the third-degree spherical-radial rule to approximate the multi-dimensional integral I(f), then:
  (16) $$I_{\mathrm{CKF}}\left(f\right)=\frac{1}{2n_{x}}\sum_{i=1}^{2n_{x}}f\left(x_{i}\right)=\frac{1}{2n_{x}}\sum_{i=1}^{2n_{x}}f\left(\bar{x}\right)+D_{\Delta x_{i}}f+\frac{D_{\Delta x_{i}}^{2}f}{2!}+\frac{D_{\Delta x_{i}}^{3}f}{3!}+\frac{D_{\Delta x_{i}}^{4}f}{4!}+\cdots$$
  where $x_{i}$ is the sampled cubature point, $\Delta x=x_{i}-\bar{x}$. Through proper simplification, the final result can be written as:
  (17) $$I_{\mathrm{CKF}}\left(f\right)=f\left(\bar{x}\right)+\left(\frac{\nabla^{T}P\nabla }{2!}\right)f+\frac{n^{k-1}}{\left(2k\right)!}{\sum_{i=1}^{n_{x}}{\left(a_{i1}\frac{\partial }{\partial x_{1}}+\cdots +a_{in}\frac{\partial }{\partial x_{n}}\right)}^{2k}f\left(x\right)|}_{x=\bar{x}}$$
  where k=1,2,3,⋯, $a_{ij}={\left[\sqrt{P}\right]}_{ij},\;i,j=1,2,\cdots ,n$, $\frac{\partial }{\partial x_{i}}f$ is the partial derivative of f(x) at the *i*th component of x. The interested reader can be referred to paper [26] for details in the extension.
(2) Like the derivation process of $I_{\mathrm{CKF}}\left(f\right)$, we use UKF to operate the approximation, and the final result is:
  (18) $$I_{\mathrm{UKF}}\left(f\right)=f\left(\bar{x}\right)+\left(\frac{\nabla^{T}P\nabla }{2!}\right)f+\frac{{\left(n+\kappa \right)}^{k-1}}{\left(2k\right)!}{\sum_{i=1}^{n_{x}}{\left(a_{i1}\frac{\partial }{\partial x_{1}}+\cdots +a_{in}\frac{\partial }{\partial x_{n}}\right)}^{2k}f\left(x\right)|}_{x=\bar{x}}$$
  where κ is the scaling parameter, and the remaining notations are the same as Equation (17). To capture the kurtosis of the prior density as correctly as possible, κ is suggested to be $\kappa =3-n_{x}$ [16].

This paper is to tackle the high-dimensional estimation problem, so here we just discuss the case in which the state dimension $n_{x}>3$. As is shown in Equations (17) and (18), the first two terms in both equations are the same, the only difference is in the last term. If $n_{x}>3$, $n^{k-1}>{\left(n+\kappa \right)}^{k-1}$, and 2*k* is an even number, then $I_{\mathrm{CKF}}\left(f\right)>I_{\mathrm{UKF}}\left(f\right)$, which indicates that CKF is more accurate than UKF in high-dimensional estimation.

We choose the stability factor $I=\sum_{i}\left|\omega_{i}\right|/\sum_{i}\omega_{i}$ defined as the measure of the numerical stability of the multi-dimensional integral, where $\omega_{i}$ is the sampling weight. It is proven that the sampling rule implemented in a finite-precision arithmetic machine introduces a large amount of round-off errors when the stability factor *I* is larger than unity [19].

In UKF, if $n_{x}>3$, and $\kappa +n_{x}=3$, so $\kappa =3-n_{x}<0$, then $\omega_{0}=1-n_{x}/3<0$. The stability factor is $I_{\mathrm{UKF}}=\frac{2n_{x}}{3}-1>1$, and it scales linearly with state dimension $n_{x}$, which indicates that UKF introduces significant perturbations in numerical estimates for the moment integral. However, in CKF, the stability factor $I_{\mathrm{CKF}}$ always meets $I_{\mathrm{CKF}}=1$, which shows that CKF is more accurate than UKF for high-dimensional state estimation.

## 4. Centralized Multi-Sensor Square Root Cubature Joint Probabilistic Data Association

In CMSCJPDA, measurements of each sensor are processed in sequence, then the multi-sensor multi-target tracking problem can be reduced to several single-sensor multi-target tracking problems, which are easier to solve.

Assume there are $m_{i,\;k}^{t}$ validated measurements for target *t* obtained by sensor *i* at time instant *k*. $l_{i}\;\left(0\leq l_{i}\leq m_{i,\;k}^{t}\right)$ is the label of validated measurements from sensor i, $l_{i}=j\;\left(j\neq 0\right)$ represents the *j*th measurements in the validated region. Especially, $l_{i}=0$ indicates that there is no measurement in the validated region. $Z_{l_{i}}^{i,t}\left(k\right)$ represents the $l_{i}$th validated measurement, ${\hat{Z}}_{i|i-1}^{t}\left(k|k\right)$ represents the predicted measurement, which will be discussed in detail later. $\beta_{l_{i},i}^{t}\left(k\right)$ represents the association probability that the measurement $l_{i}$ originated from target *t*, $K_{i}^{t}\left(k\right)$ is the filtering gain. The state estimate and the corresponding error covariance after processing the measurements of the *i*th sensor are denoted by ${\hat{X}}_{i}^{t}\left(k|k\right)$ and $P_{i}^{t}\left(k|k\right)$, respectively. ${\hat{X}}_{0}^{t}\left(k|k\right)$ and $P_{0}^{t}\left(k|k\right)$ represent the initial estimation, and ${\hat{X}}^{t}\left(k|k\right)$ and $P^{t}\left(k|k\right)$ represent the final estimation at time instant *k*, respectively. Then, the state update is as follows:

(19) $${\hat{X}}_{i}^{t}\left(k|k\right)={\hat{X}}_{i-1}^{t}\left(k|k\right)+K_{i}^{t}\left(k\right)\sum_{l_{i}=0}^{m_{i,\;k}^{t}}\beta_{i,\;l_{i}}^{t}\left(k\right)\left[Z_{l_{i}}^{i,t}\left(k\right)-{\hat{Z}}_{i|i-1}^{t}\left(k|k\right)\right]$$

where:

(20) $$\{\begin{array}{c} {\hat{X}}_{0}^{t}\left(k|k\right)={\hat{X}}^{t}\left(k|k-1\right),\;{\hat{X}}^{t}\left(k|k\right)={\hat{X}}_{N_{s}}^{t}\left(k|k\right)\\ P_{0}^{t}\left(k|k\right)=P^{t}\left(k|k-1\right),\;P^{t}\left(k|k\right)=P_{N_{s}}^{t}\left(k|k\right) \end{array}$$

Then, the update of the error covariance is:

(21) $$\begin{array}{c} P_{i}^{t}\left(k|k\right)=P_{i-1}^{t}\left(k|k\right)-\left[1-\beta_{0,i}^{t}\left(k\right)\right]K_{i}^{t}\left(k\right)S_{i}^{t}\left(k\right){\left[K_{i}^{t}\left(k\right)\right]}^{T}\\ \;+\sum_{l_{i}=0}^{m_{i,\;k}^{t}}\beta_{i,\;l_{i}}^{t}\left(k\right){\hat{X}}_{i,\;l_{i}}^{t}\left(k|k\right){\left[{\hat{X}}_{i,\;l_{i}}^{t}\left(k|k\right)\right]}^{T}-{\hat{X}}_{i}^{t}\left(k|k\right){\left[{\hat{X}}_{i}^{t}\left(k|k\right)\right]}^{T} \end{array}$$

Note that the key point for nonlinear single sensor multi-target tracking problem is to compute the association probability $\beta_{i,\;l_{i}}^{t}\left(k\right)$ and nonlinear state estimation ${\hat{X}}_{i}^{t}\left(k|k\right)$. JPDA is thought to be an effective method for multiple targets tracking with single sensor, so we choose JPDA to compute $\beta_{i,\;l_{i}}^{t}\left(k\right)$ here. The remaining problem is to obtain ${\hat{X}}_{i}^{t}\left(k|k\right)$. Although CKF is a good method to deal with the nonlinear state estimation problem, it is easily influenced by limited computer word-length and numerical errors, which may lead to loss of positive definiteness and symmetry of the error covariance matrix [19,21,22,23,24]. The effective way to preserve both properties and improve the numerical stability is to design a square root version of the CKF. Despite the fact that the square root cubature Kalman filter (SRCKF) is reformulated to propagate the square roots of the covariance matrices, both CKF and SRCKF are algebraically equivalent to each other, the interested reader is referred to [19]. In the proposed method, we choose SRCKF to update the state of the targets after the correlation step.

### 4.1. Computation of Association Probabilities

The probability $\beta_{i,\;l_{i}}^{t}\left(k\right)$ that measurement *l_i_* originated from target *t* can be calculated by summing over all feasible events for which it is true [1]:

(22) $$\beta_{l_{i},i}^{t}\left(k\right)=\sum_{m=1}^{n_{i}^{t}\left(k\right)}{\hat{\omega }}_{l_{i},\;t}^{m}\left(\theta_{m}\left(k\right)\right)\mathrm{Pr}\left\{\theta_{m}\left(k\right)|Z_{i}^{t}\left(k\right)\right\}$$

where $n_{i}^{t}\left(k\right)$ is the number of joint events, $\theta_{m}\left(k\right)$ denotes the event *m*, and ${\hat{\omega }}_{l_{i},\;t}^{m}\left(\theta_{m}\left(k\right)\right)$ is the following binary element.

(23) $${\hat{\omega }}_{l_{i},\;t}^{m}\left(\theta_{m}\left(k\right)\right)=\left\{ \begin{array}{c} 1,\;\mathrm{if}\;\theta_{m}\left(k\right)\;\mathrm{occurs}\\ 0,\;\mathrm{else} \end{array}\right.$$

$\mathrm{Pr}\left\{\theta_{m}\left(k\right)|Z_{i}^{t}\left(k\right)\right\}$ represents the posterior probability of event *m*, which can be computed by Equation (24):

(24) $$\begin{array}{c} \mathrm{Pr}\left\{\theta_{m}\left(k\right)|Z_{i}^{t}\left(k\right)\right\}=\frac{1}{C}\;\frac{\phi \left[\theta_{m}\left(k\right)\right]!}{V^{\phi \left[\theta_{m}\left(k\right)\right]}}\\ \;\cdot \prod_{l_{i}=1}^{m_{i,\;k}^{t}}N_{l_{i}}^{i,\;t}{\left[Z_{l_{i}}^{i,t}\left(k\right)\right]}^{\tau_{l_{i}}\left[\theta_{m}\left(k\right)\right]}\prod_{t=1}^{N_{t}}{\left(P_{D}^{t}\right)}^{\delta_{t}\left[\theta_{m}\left(k\right)\right]}{\left(1-P_{D}^{t}\right)}^{1-\delta_{t}\left[\theta_{m}\left(k\right)\right]} \end{array}$$

where *C* is a constant, $\phi \left[\theta_{m}\left(k\right)\right]$ is the total number of false measurements in event *m*. *V* is the volume of the entire surveillance region, and $P_{D}^{t}$ is the detection probability of target *t*. The target detection indicator $\delta_{t}\left[\theta_{m}\left(k\right)\right]$ is defined as:

(25) $$\delta_{t}\left[\theta_{m}\left(k\right)\right]=\sum_{l_{i}=1}^{m_{k_{i}}}{\hat{\omega }}_{l_{i},\;t}^{m}\left(\theta_{m}\left(k\right)\right)=\left\{ \begin{array}{c} 1,\;\mathrm{if}\;\mathrm{target}\;t\;\mathrm{is}\;\mathrm{detected}\\ 0,\;\mathrm{otherwise} \end{array}\right.$$

which indicates whether any measurement is correlated with target *t* in event *m*. In the same way, it is also easy to define measurement association indicator:

(26) $$\tau_{l_{i}}\left[\theta_{m}\left(k\right)\right]=\sum_{t=1}^{N_{t}}{\hat{\omega }}_{l_{i},\;t}^{m}\left(\theta_{m}\left(k\right)\right)=\left\{ \begin{array}{c} 1,\;\mathrm{if}\;\mathrm{measurement}\;l_{i}\;\mathrm{is}\;\mathrm{associated}\;\mathrm{with}\;a\;\mathrm{target}\\ 0,\;\mathrm{otherwise} \end{array}\right.$$

which indicates whether measurement $l_{i}$ is correlated with a target in event *m*. $N_{l_{i}}^{i,\;t}\left[Z_{l_{i}}^{i,t}\left(k\right)\right]$ is a conditional probability density function under Gaussian assumption, i.e.:

(27) $$\begin{array}{c} N_{l_{i}}^{i,\;t}\left[Z_{l_{i}}^{i,t}\left(k\right)\right]=\frac{1}{\sqrt{\left|2\pi S_{i}^{t}\left(k\right)\right|}}\exp\{-\frac{1}{2}{\left[Z_{l_{i}}^{i,t}\left(k\right)-{\hat{Z}}_{i|i-1}^{t}\left(k|k\right)\right]}^{T}\\ \cdot {\left[S_{i}^{t}\left(k\right)\right]}^{-1}\left[Z_{l_{i}}^{i,t}\left(k\right)-{\hat{Z}}_{i|i-1}^{t}\left(k|k\right)\right]\} \end{array}$$

### 4.2. State Update

During the update of state, the data processing in first sensor is a little different from that in other sensors. Firstly, the specific procedures for the state update in sensors with label i>1 are as follows:

**Step 1:** Calculate the cubature points (*j* = 1, 2, …, 2*n_x_*):

(28) $${\hat{X}}_{\;i|i-1}^{t}\left(k|k\right)={\hat{X}}_{\;i-1|i-1}^{t}\left(k|k\right)$$

(29) $$X_{j,\;i|i-1}^{t}\left(k|k\right)=S_{i|i-1}^{t}\left(k|k\right)\xi_{j}+{\hat{X}}_{i|i-1}^{t}\left(k|k\right)$$

where *n_x_* is the dimension of the state. $S_{i|i-1}^{t}\left(k|k\right)$ is the square root factor of $P_{i-1}^{t}\left(k|k\right)$, i.e.:

(30) $$P_{i-1}^{t}\left(k|k\right)=S_{i|i-1}^{t}\left(k|k\right){\left[S_{i|i-1}^{t}\left(k|k\right)\right]}^{T}$$

Equations (28) and (29) indicate that the estimated state ${\hat{X}}_{i-1|i-1}^{t}\left(k|k\right)$ and error covariance $P_{i-1}^{t}\left(k|k\right)$ of the previous sensor are regarded as the predicted state ${\hat{X}}_{i|i-1}^{t}\left(k|k\right)$ and error covariance $P_{i|i-1}^{t}\left(k|k\right)$ of the next sensor, which is the greatest difference of the sensors with label i>1 compared with the first sensor with label i=1 in the process of state estimation. That is to say, the time update is not needed in sensors with label i>1, and the estimation of the previous sensor is used as the prediction of the next sensor.

**Step 2:** Evaluate the prediction of cubature points:

(31) $$Z_{j,i|i-1}^{t}\left(k|k\right)=h^{i}\left[k,X_{j,\;i|i-1}^{t}\left(k|k\right)\right]$$

**Step 3:** Estimate the prediction of the corresponding measurement:

(32) $${\hat{Z}}_{i|i-1}^{t}\left(k|k\right)=\frac{1}{2n_{x}}\sum_{j=1}^{2n_{x}}Z_{j,\;i|i-1}^{t}\left(k|k\right)$$

**Step 4:** Estimate the square-root of the innovation covariance:

(33) $$S_{zz,\;i|i-1}^{t}\left(k|k\right)=\mathrm{Tria}\left(\left[Z_{i|i-1}^{t}\left(k|k\right)\;S_{R^{i}(k)}\right]\right)$$

where $S_{zz,\;i|i-1}^{t}\left(k|k\right)$ is a lower triangular matrix. $S_{R^{i}(k)}$ denotes a square root factor of $R^{i}\left(k\right)$ that $R^{i}\left(k\right)=S_{R^{i}(k)}{\left[S_{R^{i}(k)}\right]}^{T}$, and the weighed, centred matrix:

(34) $$Z_{i|i-1}^{t}\left(k|k\right)=\frac{1}{\sqrt{2n_{x}}}\left[Z_{1,\;i|i-1}^{t}\left(k|k\right)-{\hat{Z}}_{i|i-1}^{t}\left(k|k\right),\;Z_{2,\;i|i-1}^{t}\left(k|k\right)-{\hat{Z}}_{i|i-1}^{t}\left(k|k\right),\;\cdots ,\;Z_{2n_{x},\;i|i-1}^{t}\left(k|k\right)-{\hat{Z}}_{i|i-1}^{t}\left(k|k\right)\right]$$

Note that S=Tria(A) denotes the QR decomposition, where S is a lower triangular matrix. The relationship of matrices S and A is as follows: Let B be an upper triangular matrix obtained from the QR decomposition on $A^{T}$. Then, the lower triangular matrix S is obtained as $S=R^{T}$.

**Step 5:** Estimate the cross-covariance matrix:

(35) $$P_{xz,i|i-1}^{t}\left(k|k\right)=X_{i|i-1}^{t}\left(k|k\right){\left[Z_{i|i-1}^{t}\left(k|k\right)\right]}^{T}$$

where:

(36) $$X_{i|i-1}^{t}\left(k|k\right)=\frac{1}{\sqrt{2n_{x}}}\left[X_{1,\;i|i-1}^{t}\left(k|k\right)-{\hat{X}}_{i|i-1}^{t}\left(k|k\right),X_{2,\;i|i-1}^{t}\left(k|k\right)-{\hat{X}}_{i|i-1}^{t}\left(k|k\right),\;\cdots ,\;X_{2n_{x},\;i|i-1}^{t}\left(k|k\right)-{\hat{X}}_{i|i-1}^{t}\left(k|k\right)\right]$$

**Step 6:** Estimate the filter gain:

(37) $$K_{i}^{t}\left(k\right)={P_{xz,i|i-1}^{t}\left(k|k\right)/S_{zz,\;i|i-1}^{t}\left(k|k\right)/\left[S_{zz,\;i|i-1}^{t}\left(k|k\right)\right]}^{T}$$

**Step 7:** Update the nonlinear state:

(38) $${\hat{X}}_{l_{i},\;i}^{t}\left(k|k\right)={\hat{X}}_{i-1}^{t}\left(k|k\right)+K_{i}^{t}\left(k\right)\left[Z_{l_{i}}^{i,t}\left(k\right)-{\hat{Z}}_{i|i-1}^{t}\left(k|k\right)\right]$$

Equations (28)–(38) give a solution to the estimation of the target state and error covariance at time instant *k* with sensor label i>1.

Now the estimation problem in the first sensor with label i=1 is considered, the process of the measurement update is the same as that in sensors with label i>1, but the time update is quite different. In the sensor with label i=1, the estimation of prediction of the state and error covariance is essential. The current cubature points are:

(39) $$X_{j}^{t}\left(k-1|k-1\right)=S^{t}\left(k-1|k-1\right)\xi_{j}+{\hat{X}}^{t}\left(k-1|k-1\right)$$

The propagated cubature points are:

(40) $$X_{j}^{t}\left(k|k-1\right)=f^{t}\left[k-1,X_{j}^{t}\left(k-1|k-1\right)\right]$$

Then the predicted state is:

(41) $${\hat{X}}^{t}\left(k|k-1\right)=\frac{1}{2n_{x}}\sum_{j=1}^{2n_{x}}X_{j}^{t}\left(k|k-1\right)$$

and the predicted error covariance is:

(42) $$\begin{array}{c} P^{t}\left(k|k-1\right)=\frac{1}{2n_{x}}\sum_{j=1}^{2n_{x}}\left[X_{j}^{t}\left(k|k-1\right)-{\hat{X}}^{t}\left(k|k-1\right)\right]\\ \cdot {\left[X_{j}^{t}\left(k|k-1\right)-{\hat{X}}^{t}\left(k|k-1\right)\right]}^{T}+Q^{t}\left(k\right) \end{array}$$

Equations (39)–(42) describe the time update in the first sensor with label i=1. With the measurement update process composed of Equations (28)–(38) applied, the estimated state ${\hat{X}}_{1}^{t}\left(k|k\right)$ and error covariance $P_{1}^{t}\left(k|k\right)$ can be achieved respectively after the data of the first sensor is processed.

To state the proposed method more clearly, the algorithm flow of CMSCJPDA is illustrated in Table 1.

## 5. Numerical Experiments

In this section, the numerical results and the effectiveness of the proposed CMSCJPDA algorithm are discussed in two simulation scenarios: crossing target tracking and maneuvering target tracking. The performance of CMSCJPDA is compared with MSJPDA-EKF and MSJPDA-UKF in two scenarios.

For a fair comparison, we make 50 independent Monte Carlo runs. In each run, the measurement noises are generated independently, and the corresponding noisy position measurements are used to initialize the estimate, which guarantees consistency of the initialization of the filter and makes the final estimate unbiased. Except for the initialization, the process of each Monte Carlo run is the same, and the mean value of 50 runs is considered as the final result. The total number of steps per run is 100. T=1 s is the sampling interval. The nonparametric model is used for the probability mass function (PMF) of the number of false measurements. The expected number of false measurements in the validation gate is m=2. The detection probabilities of all targets are assumed to be $P_{D}=0.9$. The probability mass is assumed to be $P_{G}=0.9997$. Due to the large initial error, we display the numerical results after the tenth step for clarity.

Targets in the surveillance area are observed by three two-dimensional sensors. The measurement equation of sensor i is:

(43) $$Z^{i}\left(k\right)=\left[\begin{array}{c} \sqrt{{\left(x(k)-x_{pi}\right)}^{2}+{\left(y(k)-y_{pi}\right)}^{2}}\\ \arctan\left(\frac{y(k)-y_{pi}}{x(k)-x_{pi}}\right) \end{array}\right]+W^{i}\left(k\right)$$

where i=1,2,3 is the sensor label. (x(k), y(k)) is the true state of the targets, $\left(x_{pi},\;y_{pi}\right)$ is the position of sensor *i*. Other parameters of the sensors are set as shown in Table 2.

*Performance metrics*: To compare the tracking performance of different algorithms, the root mean square error (RMSE) is chosen as the metric. The root mean square position error of target *t* at time instant *k* is defined as:

(44) $${\mathrm{RMSE}}_{\mathrm{pos}}\left(k,\;t\right)=\sqrt{\frac{1}{M}\sum_{i=1}^{M}{\left(x_{i}^{t}\left(k\right)-{\hat{x}}_{i}^{t}\left(k|k\right)\right)}^{2}}$$

where M is the total number of Monte Carlo simulations. $x_{i}^{t}\left(k\right)$ and ${\hat{x}}_{i}^{t}\left(k|k\right)$ are true and estimated positions of target *t* in the *x* direction in the *n*-th Monte Carlo run. Similarly, we may also define formulas of the root mean square velocity error.

### 5.1.Crossing Targets Tracking

#### 5.1.1. Simulation Scenario

Consider a two-crossing target tracking case. The state model is:

(45) X(k+1)=F(k)X(k)+Γ(k)V(k)

where the component of process noise $q_{1}=q_{2}=0.01$. The state transition matrix F(k) is defined as:

(46) $$F\left(k\right)=\left[\begin{array}{cccc} 1 & T & 0 & 0\\ 0 & 1 & 0 & 0\\ 0 & 0 & 1 & T\\ 0 & 0 & 0 & 1 \end{array}\right]$$

and the process noise distribution matrix is:

(47) $$\Gamma \left(k\right)=\left[\begin{array}{cc} T^{2}/2 & 0\\ T & 0\\ 0 & T^{2}/2\\ 0 & T \end{array}\right]$$

The initial states of two targets are ***X***_1_(0) = [−29,500 m, 400 m/s, 34,500 m, 2,212,400 m/s]^T^, ***X***_2_(0) = [−26,500 m, 296 m/s, 34,500 m, −400 m/s]^T^. After about 31 s, the two targets cross at the point (−17,000 m, 22,000 m).

#### 5.1.2. Results and Analysis

**Figure 1:**
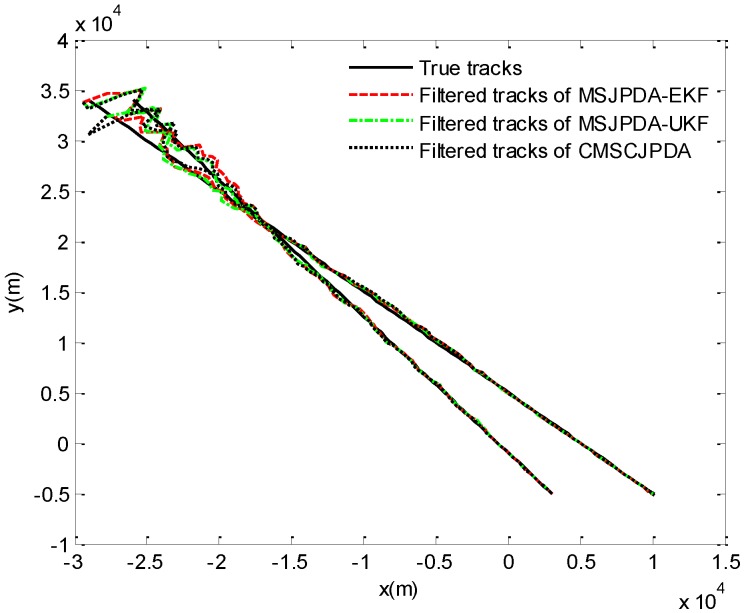
True tracks and filtered tracks of two crossing targets.

The true and filtered tracks of two crossing targets are illustrated in Figure 1, which shows the tracking performance of three algorithms in a single run.

**Figure 2:**
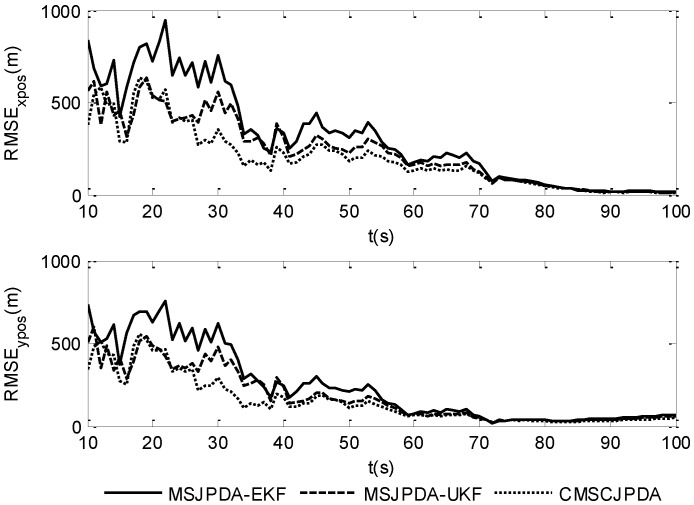
Root mean square position error of target 1.

**Figure 3:**
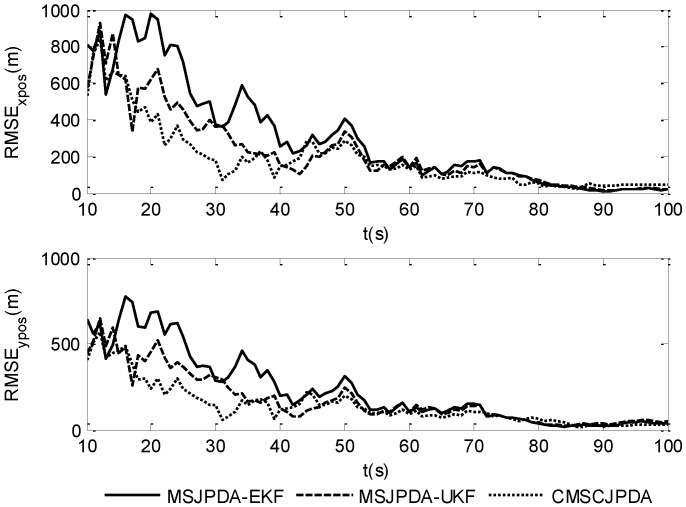
Root mean square position error of target 2.

**Figure 4:**
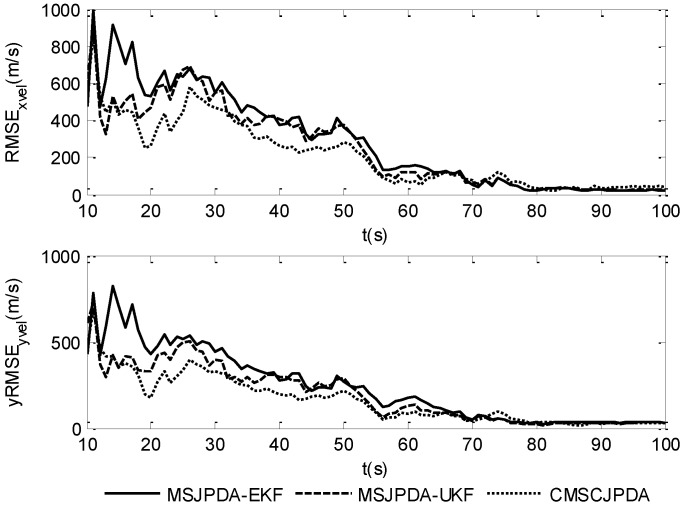
Root mean square velocity error of target 1.

**Figure 5:**
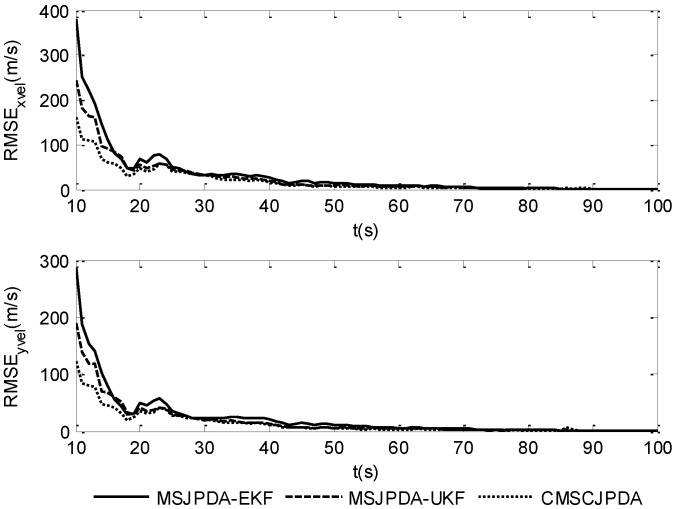
Root mean square velocity error of target 2.

To give a more explicit picture of the estimated results, the root mean square position error of three data association algorithms in the *x* and *y* directions is shown in Figure 2 and Figure 3, while the root mean square velocity error in the *x* and *y* directions is shown in Figure 4 and Figure 5. As is shown, the three algorithms can effectively estimate the state of both targets. However, CMSCJPDA and MSJPDA-UKF, which are based on deterministic sampling, are of higher tracking accuracy than MSJPDA-EKF, and CMSCJPDA is slightly more accurate than MSJPDA-UKF, which just validates the argument in [19]. With the square root version of CKF applied in the proposed method, the square rooting operations of covariance matrix is avoided and the tracking performance is effectively enhanced.

To further validate the effectiveness of the proposed algorithm, numerical stability and computational complexity of the three algorithms are compared in Table 3, which gives the average divergence times (ADT) and average time consumption (ATC) in 50 Monte Carlo runs. As is shown, the ADT of MSJPDA-EKF is twice more than that of MSJPDA-UKF, and nearly four times more than that of CMSCJPDA, to some extent CMSCJPDA is relatively more stable in terms of convergence. As for the computational cost, the ATC of CMSCJPDA is almost half that of MSJPDA-EKF, and less than that of MSJPDA-UKF. In a word, compared with MSJPDA-EKF and MSJPDA-UKF, CMSCJPDA can achieve relatively higher tracking accuracy, and is more stable and less computationally complex under a nonlinear cluttered environment.

### 5.2. Maneuvering Targets Tracking

#### 5.2.1. Simulation Scenario

**Figure 6:**
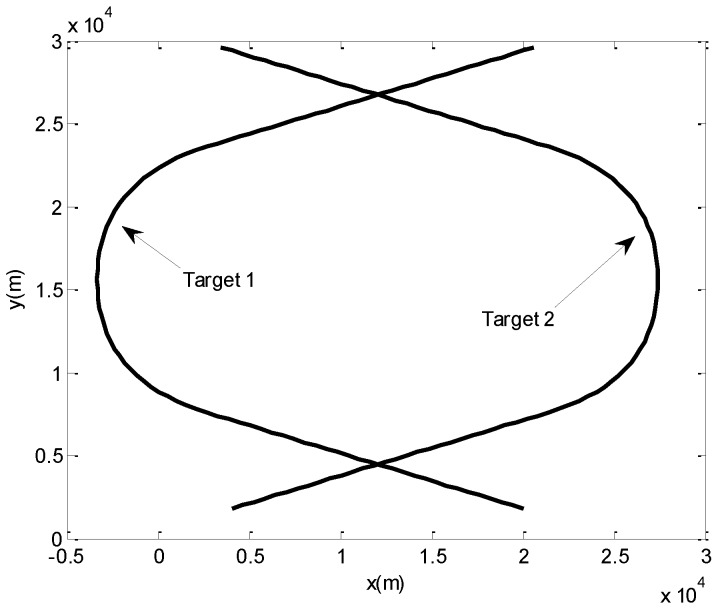
The trajectories of two cross-maneuvering targets.

Assume there are two maneuvering targets in the surveillance area. The initial states of two targets are *X*_1_(0) = [20,000 m, −600 m/s, 1800 m, 500 m/s]^T^, *X*_2_(0) = [4000 m, 600 m/s,1800 m, 200 m/s]^T^. The two targets move at a constant speed in a straight line for 30 s. Then, from 30 s to 50 s, they both move with constant accelerations of *a*_1*x*_ = −30 m/s^2^, *a*_1*y*_ = 20 m/s^2^, *a*_2*x*_ = 30 m/s^2^, *a*_2*y*_ = 20 m/s^2^ in the *x* and *y* directions, respectively. From 50 s to 70 s, they still move with constant accelerations, and the accelerations for both targets in the *x* and *y* directions are *a_x_*_1_ = −30 m/s^2^, *a_y_*_1_ = −20 m/s^2^, *a_x_*_2_ = 30 m/s^2^, *a_y_*_2_ = −20 m/s^2^, respectively. After 70 s, they both move at a constant speed again until 100 s. The true trajectories of both targets are illustrated in Figure 6.

#### 5.2.2. Results and Analysis

**Figure 7:**
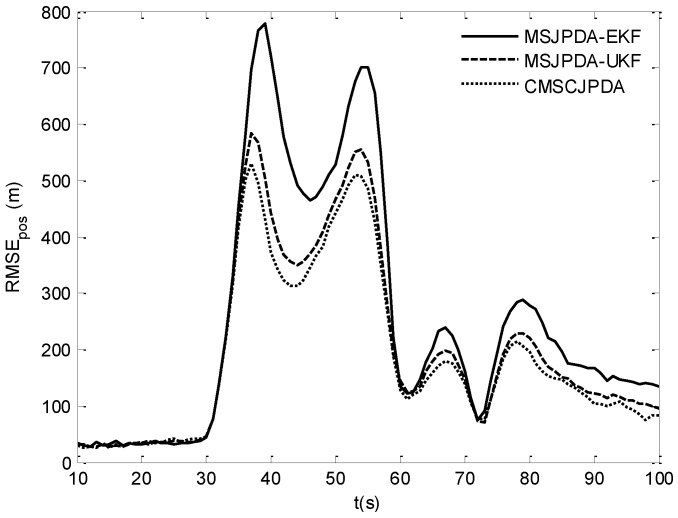
Root mean square position error of target 1.

**Figure 8:**
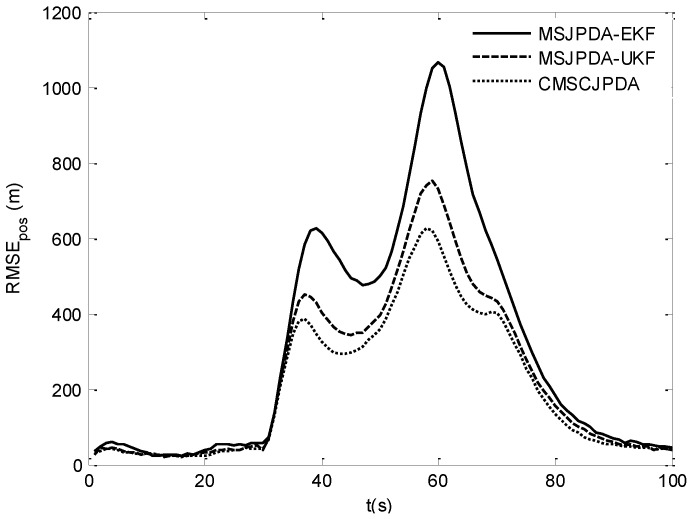
Root mean square position error of target 2.

The root mean square (RMS) position error of two targets is compared in Figure 7 and Figure 8. The results show that the estimation error of CMSCJPDA and MSJPDA-UKF is much smaller than that of MSJPDA-EKF in the whole simulation. When *t* is about 40 s, the estimation error of MSJPDA-EKF is about 200 m larger compared with CMSCJPDA and MSJPDA-UKF. Moreover, with the new association strategy for maneuvering target tracking, CMSCJPDA has relatively higher accuracy than MSJPDA-UKF. At some time instants, the RMS position error of CMSCJPDA is about 50 m smaller than that of MSJPDA-UKF, and it converges relatively faster.

Table 4 evaluates the tracking performance of the three algorithms in the aspects of mean RMS position error (MRMSE) and correct association rate (CAR). The results indicate that both CMSCJPDA and MSJPDA-UKF are more accurate than MSJPDA-UKF, and CMSCJPDA is slightly more accurate than MSJPDA-UKF. Meanwhile, the CAR of CMSCJPDA is the highest among the three algorithms, which is 11.1% higher than MSJPDA-UKF and 28.7% higher than MSJPDA-EKF, and CMSCJPDA is more time-saving.

## 6. Conclusions

In this paper, we propose a centralized multi-sensor square root cubature joint probabilistic data association algorithm, which is based on the spherical-radial principle and the sequential update scheme. Compared with the state-of-the-art method in two tracking scenarios, the proposed method is much more accurate than MSJPDA-EKF, and a slightly better than MSJPDA-UKF by using the square root version of cubature Kalman filter to estimate the target state. Furthermore, the experimental results also show that the proposed method is less prone to divergence than MSJPDA-EKF and MSJPDA-UKF, and is more stable with respect to its numerical characteristics. Although the proposed method is less time consuming among the compared methods, due to complicated calculation of the association probability, the joint probabilistic data association-based methods are of significant computational cost, which is still less practical in real applications. Currently, the sensors in our experiment are assumed to be synchronized sampling, and there is no time difference in measurements from different sensors. However, the actual applications can rarely meet this condition, and in the future we will pay more attention to reducing the computational cost and improving the real-time performance. At the same time, the tracking problem with asynchronous sampling and changing the number of targets also deserves consideration.

## Figures and Tables

**Table 1:** Algorithm flow of CMSCJPDA.

**Input:** The estimated state ${\hat{X}}^{t}\left(k-1|k-1\right)$ and error covariance $P^{t}\left(k-1|k-1\right)$ at time instant k−1; **Output:** The estimated state ${\hat{X}}^{t}\left(k|k\right)$ and error covariance $P^{t}\left(k|k\right)$ at time instant k;
**Step 1.** Time update (i=1) Compute the predicted state ${\hat{X}}^{t}\left(k|k-1\right)$ and error covariance $P^{t}\left(k|k-1\right)$ according to Equations (39)–(42); **Step 2.** Initialization ${\hat{X}}_{0}^{t}\left(k|k\right)={\hat{X}}^{t}\left(k|k-1\right)$, $P_{0}^{t}\left(k|k\right)=P^{t}\left(k|k-1\right)$; **Step 3.** Measurement update For $t=1:N_{t}$ For $i=1:N_{s}$ Compute the estimation of state ${\hat{X}}_{i}^{t}\left(k|k\right)$ and error covariance $P_{i}^{t}\left(k|k\right)$ with the validated measurements of sensor i according to Equations (19)–(38); End End **Step 4.** Establish the final estimation ${\hat{X}}^{t}\left(k|k\right)={\hat{X}}_{N_{s}}^{t}\left(k|k\right)$, $P^{t}\left(k|k\right)=P_{N_{s}}^{t}\left(k|k-1\right)$

**Table 2:** Sensor position and parameter setting.

Sensor Labe	Sensor Position (m)	Ranging Error (m)	Angle Error (rad)
1	(0, 0)	100	0.01
2	(−500, −500)	200	0.02
3	(−500, 500)	300	0.03

**Table 3:** Performance comparison of the three algorithms.

Algorithms	Average Divergence Times	Average Time Consumption (s)
MSJPDA-EKF	0.87	0.423
MSJPDA-UKF	0.42	0.346
CMSCJPDA	0.27	0.257

**Table 4:** Performance comparison of three algorithms.

Algorithms	MRMSE (m)	CAR (%)	TC (s)
MSJPDA-EKF	206.2	47.6	0.735
MSJPDA-UKF	132.6	65.2	0.563
CMSCJPDA	104.3	76.3	0.432

## Appendix A. Proof of Equation (14)

For simplicity, define the weighting function $\omega_{1}\left(x\right)=N\left(x;\;0,\;I\right)$ and $\omega_{2}\left(x\right)=N\left(x;\;\mu ,\;\Sigma \right)$. Consider the left side of Equation (14). Since Σ is a positive definite matrix, then Σ can be factorized to be $\Sigma =\sqrt{\Sigma }{\left(\sqrt{\Sigma }\right)}^{T}$. Making a variable substitution by $x=\sqrt{\Sigma }y+\mu$, we have:

(A1) $$\begin{array}{cc} \int_{R^{n}}f\left(x\right)\omega_{2}\left(x\right)dx & =\int_{R^{n}}f\left(\sqrt{\Sigma }y+\mu \right)\frac{1}{\sqrt{\left|2\pi \Sigma \right|}}\exp\left\{-{\left(\sqrt{\Sigma }y\right)}^{T}\Sigma^{-1}\left(\sqrt{\Sigma }y\right)\right\}\left|\sqrt{\Sigma }\right|dy\\ & =\int_{R^{n}}f\left(\sqrt{\Sigma }y+\mu \right)\frac{1}{\sqrt{\left|2\pi I\right|}}\exp\left(-y^{T}y\right)dy\\ & =\int_{R^{n}}f\left(\sqrt{\Sigma }y+\mu \right)N\left(y;\;0,\;I\right)dy\\ & =\int_{R^{n}}f\left(\sqrt{\Sigma }x+\mu \right)\omega_{1}\left(x\right)dx \end{array}$$

Substituting $\omega_{1}\left(x\right)$ and $\omega_{2}\left(x\right)$ into Equation (A1), we have:

(A2) $$\int_{R^{n}}f\left(x\right)N\left(x;\;\mu ,\;\Sigma \right)dx=\int_{R^{n}}f\left(\sqrt{\Sigma }x+\mu \right)N\left(x;\;0,\;I\right)dx$$
